# Evaluating the Prevalence of Foodborne Pathogens in Livestock Using Metagenomics Approach

**DOI:** 10.4014/jmb.2109.09038

**Published:** 2021-10-15

**Authors:** Hyeri Kim, Jin Ho Cho, Minho Song, Jae Hyoung Cho, Sheena Kim, Eun Sol Kim, Gi Beom Keum, Hyeun Bum Kim, Ju-Hoon Lee

**Affiliations:** 1 Department of Animal Resources Science, Dankook University, Cheonan 31116, Republic of Korea; 2 Division of Food and Animal Science, Chungbuk National University, Cheongju 28644, Republic of Korea; 3 Division of Animal and Dairy Science, Chungnam National University, Daejeon 34134, Republic of Korea; 4 Department of Food Animal Biotechnology, Department of Agricultural Biotechnology, Center for Food and Bioconvergence, Seoul National University, Seoul 08826, Republic of Korea

**Keywords:** Foodborne-pathogens, food safety, livestock, metagenomics, 16S rRNA gene

## Abstract

Food safety is the most important global health issue due to foodborne pathogens after consumption of contaminated food. Foodborne bacteria such as *Escherichia coli*, *Salmonella enterica*, *Staphylococcus aureus*, *Campylobacter* spp., *Bacillus cereus*, *Vibrio* spp., *Yersinia enterocolitica* and *Clostridium perfringens* are leading causes of the majority of foodborne illnesses and deaths. These foodborne pathogens often come from the livestock feces, thus, we analyzed fecal microbial communities of three different livestock species to investigate the prevalence of foodborne pathogens in livestock feces using metagenomics analysis. Our data showed that alpha diversities of microbial communities were different according to livestock species. The microbial diversity of cattle feces was higher than that of chicken or pig feces. Moreover, microbial communities were significantly different among these three livestock species (cattle, chicken, and pig). At the genus level, *Staphylococcus* and *Clostridium* were found in all livestock feces, with chicken feces having higher relative abundances of *Staphylococcus* and *Clostridium* than cattle and pig feces. Genera *Bacillus*, *Campylobacter*, and *Vibrio* were detected in cattle feces. Chicken samples contained *Bacillus*, *Listeria*, and *Salmonella* with low relative abundance. Other genera such as *Corynebacterium*, *Streptococcus*, *Neisseria*, *Helicobacter*, *Enterobacter*, *Klebsiella*, and *Pseudomonas* known to be opportunistic pathogens were also detected in cattle, chicken, and pig feces. Results of this study might be useful for controlling the spread of foodborne pathogens in farm environments known to provide natural sources of these microorganisms.

## Introduction

Foodborne illness represents a major threat to public health and is frequently attributed to pathogenic microorganisms in livestock feces. Leading causes of the majority of foodborne illnesses and deaths include foodborne pathogens, such as *Escherichia coli*, *Salmonella enterica*, *Staphylococcus aureus*, *Campylobacter* spp., *Bacillus cereus*, *Vibrio* spp., *Yersinia enterocolitica* and *Clostridium perfringens* [1, 2]. In South Korea, Food and Drug Safety institute reported the number of foodborne illness cases in the past seven years (2012-2019). Although the number of outbreaks has fluctuated from year to year, there has been a general increasement. Especially, there was an increase of 29.3% in the number of outbreaks (697 cases) in 2018 compared to that in the previous year (539 cases) in South Korea.

Foodborne illness outbreaks could be attributed to insufficient heating of food and cross-contamination during the food supply process. Especially, contamination of foodborne pathogens can occur from a farm environment, such as livestock feces throughout the processing of abattoirs and packaging to markets or restaurants. Foodborne pathogens originated from livestock feces can grow in a contaminated food at any point along the food supply process [3-5]. Therefore, the understanding the public health risk of microorganisms in livestock feces is essential process for the prevention of foodborne illness. The initial step to access the public health risk of microorganisms in livestock feces is to identify and describe microbial communities including foodborne pathogens. Conventional methods to identify bacterial pathogens from various sources depend on culturing on agar plates. However, these method takes 2-3 days to culture pathogens and up to more than a week for the confirmation of specific pathogenic microorganisms [6]. Currently, metagenomics sequencing techniques as culture-independent methods are widely used to identify and characterize microbiota [7]. Therefore, we hypothesized that the metagenomics approach could be used to detect foodborne pathogens in samples such as foods, feces, and soils [8, 9].

The purpose of this study was to investigate the prevalence of foodborne pathogens in the livestock feces using the metagenomics approach.

## Materials and Methods

### Sample Collection

The feces (Cattle = 33, chicken=33, Pig=34) from the livestock were collected in South Korea. Samples were stocked immediately into -80°C deep freezer to maintain stability of microbiota from the feces (Table S1).

### DNA Extraction

Total DNA representing microbial community were extracted from 200 mg of each feces sample by using QIAamp FAST DNA Stool Mini kit (Qiagen, Germany) according to the manufacturer’s instructions with some modifications. Briefly, cell lysis was conducted with bead-beating the samples twice for 2 min at 300 rpm instead of vortex during the DNA extraction process. The isolated DNA were quantified using a Colibri Microvolume Spectrometer (Titertek Berthold, Germany) and processed further analysis.

### 16s rRNA Gene Sequencing

Fifty ng of the total DNA from each feces sample was used to amplified the V5 to V6 [10] hypervariable regions of the 16S rRNA genes using the universal primer sets 799F (5’-CMGGATTAGATACCCKGG-3’) and 1114R (5’-GGGTTGCGCTCGTTGC-3’) [11]. The PCR products were purified using the Wizard SV Gel and PCR Clean-Up System (Promega Corp., USA). The 16S rRNA gene amplicons were sequenced using the Illumina MiSeq platform at Macrogen Inc. (Korea) according to the manufacturer’s instructions.

### Bioinformatics Analysis

All the sequence data from the Illumina Miseq platform with length of less than 200 base pair were trimmed to minimize the effects of random sequencing errors. Chimeric sequences were identified and eliminated from the sequences using the UCHIME algorithm implemented in Quantitative Insights into Microbial Ecology (QIIME)[12]. The QIIME pipeline was used to conduct Operational taxonomic unit (OTU) picking, taxonomic assignment, diversity analysis, and visualization. OTU picking was performed using de novo operational taxonomic unit (OTU) clustering with an OTU definition at an identity cutoff of 97% [13]. Taxonomic assignment and classification were performed using the Ribosomal Database Project (RDP) classifier implemented in QIIME [14].

Microbial alpha diversity including Chao1, observed OTUs, Shannon and Simpson indices were calculated using QIIME. Beta-diversity was measured using both weighted and unweighted UniFrac distance metrics using QIIME [15]. Principal coordinate analysis (PCoA) plots were generated based on the weighted and unweighted UniFrac distance metrics. Analysis of similarities (ANOSIM) was used to determine whether the microbial compositions between the three groups were significantly different using QIIME and was based on the weighted and unweighted UniFrac distance metrics. The abundance of taxa among the fecal samples was compared using STAMP software [16]. Samples were compared by ANOVA, followed by the Tukey–Kramer post hoc test (*p* < 0.05).

## Results

### Sequencing Data

16S rRNA gene amplicons from cattle, chicken, and pig feces were sequenced using an Illumina MiSeq platform. As a result, a total of 12,764,596 DNA sequence reads were generated from animal feces after quality-filtering. The average number of sequences generated per feces was 118,250 for cattle, 122,438 for chicken, and 142,503 for pig (Table S2).

### Alpha Diversity

OTUs were analyzed for each sample at 97% identity to compare bacterial diversity and species richness between animal feces samples. While observed OTUs, Chao, and Shannon indices indicate the different number of bacterial species present in each livestock species, similar Simpson diversity indices among the livestock species indicate the comparable population size of each of the bacterial species present in three livestock species because Simpson index considers species evenness more than species richness (Table 1).

### Beta Diversity

**Fig. 1:**
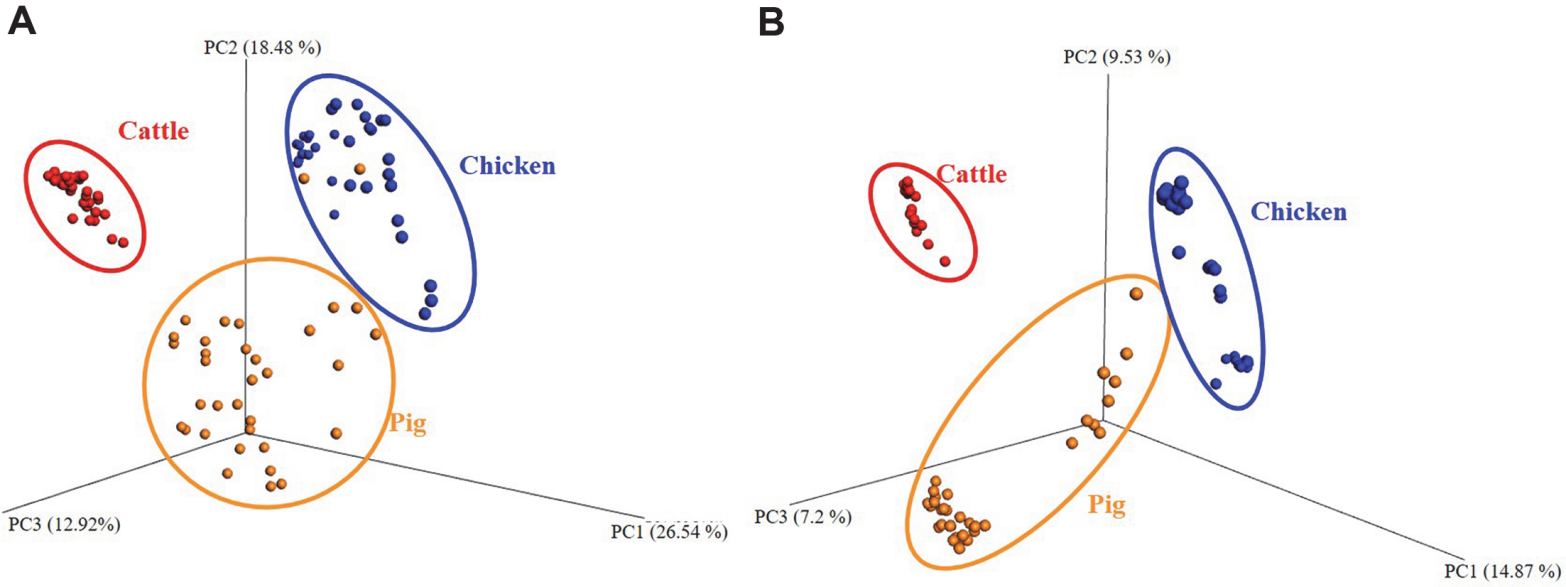
Bacterial communities using Principal coordinate analysis (PCoA) plots based on (**A**) weighted and (**B**) unweighted UniFrac distance metrics. A three-dimensional PCoA plot of the Illumina sequencing data from animal feces (*n* = 100) was generated using the software package QIIME. Samples associated with cattle (clustered by the red ellipse), chicken (clustered by the blue ellipse), and pig (clustered by the yellow ellipse) are shown as single points.

Analysis of similarities using ANOSIM of unweighted UniFrac distances indicated that samples were significantly different (*p* < 0.05) with a relatively high R-value of 0.6936, indicating that three species consisted of different microbiota. Weighted UniFrac distances from ANOSIM analysis also confirmed that distances of microbiota were similar to unweighted UniFrac distances, suggesting that microbial communities of the three species were significantly different (*p* < 0.05) with an R-value of 0.5181. PCoA plot of unweighted UniFrac visually established separated distances among the three livestock species samples (Fig. 1).

### Relative Abundance

**Fig. 2:**
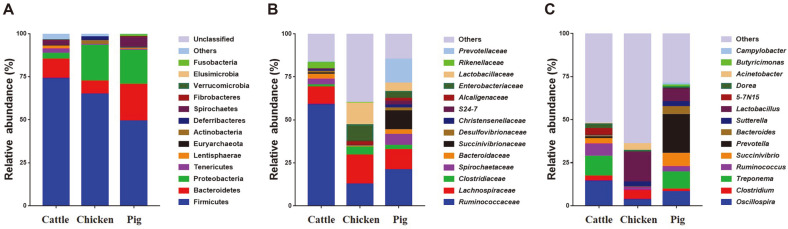
Relative abundance of taxonomy at the (**A**) phylum, (**B**) family and (**C**) genus levels in feces of cattle, chicken and pig. Phylogenetic assignment and classification based on 16S rRNA sequence similarity were conducted by using the Ribosomal Database Project (RDP) classifier implemented in QIIME.

A total of 37 phyla, 80 classes, 349 families, and 771 genera were identified in feces of these three different livestock species. Relative abundances of fecal microbiota of each livestock species at Phylum, Family, and Genus levels are shown in Fig. 2.

At the Phylum level, Firmicutes had the highest relative abundance in Cattle (73.87%), followed by that in Chicken (64.80) and Pig (49.25%) fecal microbiota. Bacteroidetes was the second abundant bacterial phylum (11.37%) in cattle fecal microbiota. Phyla Proteobacteria (20.83%), Bacteroidetes (7.64%), Deferribacteres (2.25%), and Actinobacteria (2.22%) were detected in chicken feces. The pig fecal microbial community had phyla Bacteroidetes (21.21%), Proteobacteria (19.75%), and Spirochaetes (5.71%) (Fig. 2A).

At the family level, cattle fecal microbiota mainly had *Ruminococcaceae* (58.86%), *Lachnospiraceae* (10.19%), *Rikenellaceae* (3.62%), *Spirochaetaceae* (3.27%), *Bacteroidaceae* (2.74%), and *Clostridiaceae* (1.28%). Chicken samples were predominantly composed of *Lachnospiraceae* (16.89%), *Lactobacillaceae* (12.86%), *Ruminococcaceae* (12.59%), *Enterobacteriaceae* (9.65%), *Clostridiaceae* (4.70%), and *Alcaligenaceae* (2.08%). Families *Ruminococcaceae* (20.79%), *Prevotellaceae* (13.93%), *Lachnospiraceae* (11.77%), *Succinivibrionaceae* (10.98%), *Spirochaetaceae* (6.19%), *Lactobacillaceae* (4.70%), *Enterobacteriaceae* (3.89%), *Bacteroidaceae* (2.85%), *Clostridiaceae* (2.59%), and S24-7 (2.15%) (Fig. 2B) were detected in pig fecal samples.

At the genus level, bacterial communities of different livestock species were composed of a variety of genera. *Oscillospira* and *Treponema* were the top two most significantly enriched genera in cattle fecal samples. *Lactobacillus* was the largest relative abundant genus in chicken fecal samples. *Prevotella* and *Treponema* were the most abundant genera in pig fecal samples (Fig. 2C).

**Fig. 3:**
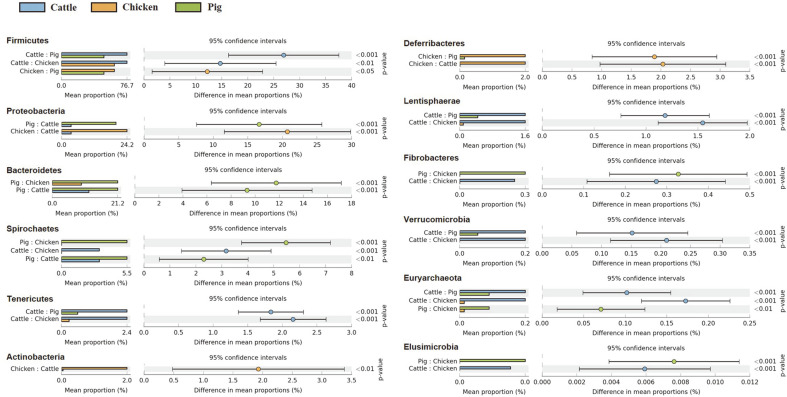
Extended error plots identifying significantly different taxa at the phylum level. Samples were compared by ANOVA, followed by the Tukey–Kramer post hoc test without correction for multiple comparisons. Corrected *p*-Values are shown at the right.

**Fig. 4:**
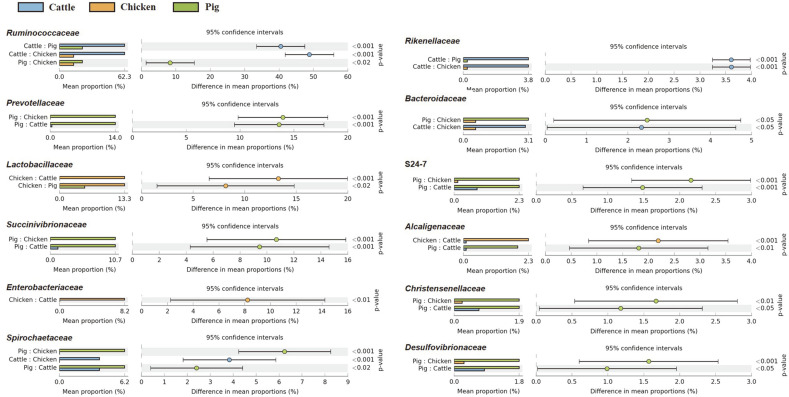
Extended error plots identifying significantly different taxa at the family level. Samples were compared by ANOVA, followed by the Tukey–Kramer post hoc test without correction for multiple comparisons. Corrected *p*-Values are shown at the right.

**Fig. 5:**
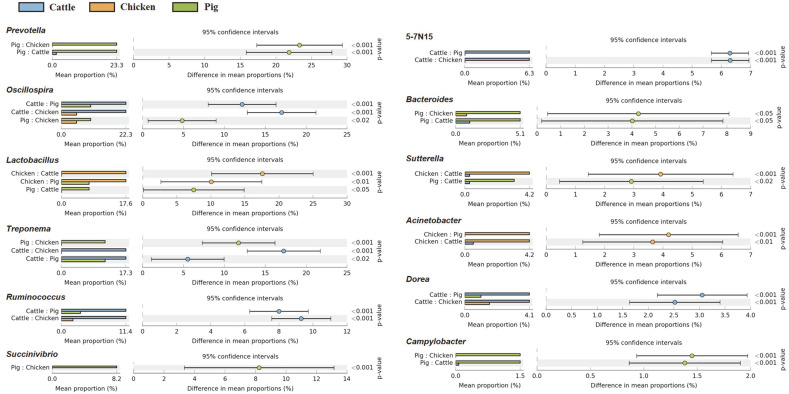
Extended error plots identifying significantly different taxa at the genus level. Samples were compared by ANOVA, followed by the Tukey–Kramer post hoc test without correction for multiple comparisons. Corrected *p*-Values are shown at the right.

Extended error bar plots were used to identify significantly different taxa at phylum, family, and genus levels among different fecal samples. At the phylum level, relative abundances of Firmicutes, Tenericutes, Lentisphaerae, Verrucomicrobia, and Euryarchaeota in cattle feces were significantly higher than those in the other two livestock species. In chicken feces, relative abundances of Proteobacteria, Actinobacteria, and Deferribacteres were statistically higher than those in other livestock species. In pig fecal samples, populations of Bacteroidetes and Spirochaetes were significantly larger than those in cattle and chicken fecal samples (Fig. 3). At the family level, relative abundances of *Ruminococcaceae* and *Rikenellaceae* were predominantly higher in cattle fecal samples than in chicken and pig fecal samples. Families *Prevotellaceae*, *Succinivibrionaceae*, *Spirochaetaceae*, S24-7, *Christensenellaceae*, and *Desulfovibrionaceae* were differentially abundant in pig feces than in other livestock species. However, *Lactobacillaceae* was the only family whose relative abundance was significantly higher in chicken feces than in the other two species (Fig. 4). At the genus level, *Oscillospira*, *Treponema*, *Ruminococcus*, 5-7N15, and Dorea showed higher abundances in cattle feces than in chicken and pig feces. Relative abundances of *Prevotella*, *Bacteroides*, and *Campylobacter* were overwhelmingly higher in pig feces than in the other two species. *Lactobacillus* and *Acinetobacter* were more detected in chicken feces than in the other two (Fig. 5).

### Detection of Foodborne Pathogens in Three Different Livestock Feces

**Fig. 6:**
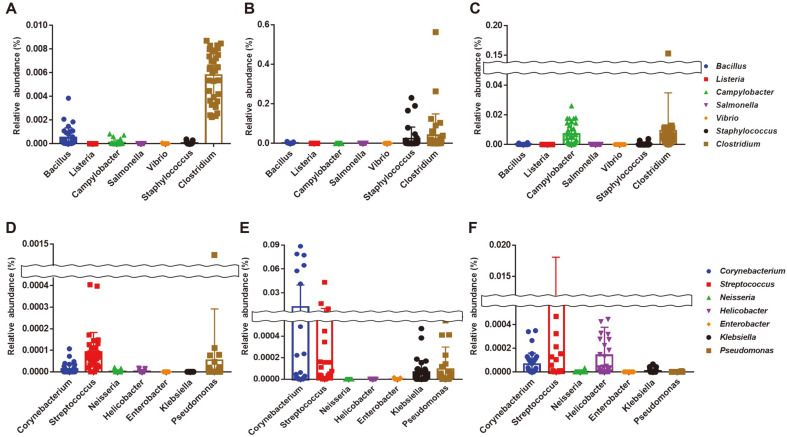
Bacterial monitoring which can be related with foodborne illness (A, B, and C) and potential pathogens (D, E, and F) in feces samples. Monitoring for foodborne pathogens were classified 5 of genera and 2 of species in feces samples from (**A**) cattle, (**B**) chickens and (**C**) pigs. Monitoring of potential pathogens which can be opportunity pathogens were also conducted in feces samples from (**D**) cattle, (**E**) chickens and (**F**) pigs.

In this study, we evaluated the prevalence of foodborne pathogens in livestock feces using the metagenomics approach. A total of 7 foodborne pathogens were detected in feces samples using 16S rRNA gene analysis. *Staphylococcus* and *Clostridium* were present in cattle, chicken, and pig feces. However, higher relative abundances of those genera were detected in chicken feces than in cattle and pig samples. In cattle feces, genera *Bacillus*, *Campylobacter*, and *Vibrio* were confirmed (Fig. 6A). *Bacillus*, *Listeria*, and *Salmonella* with low relative abundances were found in chicken feces (Fig. 6B). In pig feces, it was confirmed that *Campylobacter* had the highest relative abundance among detected foodborne pathogens. *Salmonella* was also detected (Fig. 3C). In addition, potential pathogens such as *Corynebacterium*, *Streptococcus*, *Klebsiella*, and *Pseudomonas* were confirmed in cattle, chicken, and pig feces (Figs. 3D, 3E, and 3F). Relative abundances of *Streptococcus* and *Corynebacterium* in chicken feces were higher than those in cattle and pig feces (Fig. 3E). *Helicobacter* was detected more in pig feces than in the other two (Fig. 3F).

## Discussions

This study was conducted to evaluate contamination levels of foodborne pathogens in feces of livestock animals such as cattle, chicken, and pig. It has been reported that some of pathogens, such as *Salmonella* and *Campylobacter* are asymptomatically carried in the animal’s intestinal tract. They can be shed in feces in large populations and be transmitted by other vectors from feces to humans [17]. Foodborne pathogens may cause contamination of food not only by careless food management during the entire cooking process, but also by other food supply processes ranging from the production of livestock to the distribution process. It has been reported that agricultural products might be contaminated when livestock manure is used in soil as a raw material for compost [3-5, 18].

In this study, the presence or absence of foodborne pathogens was evaluated using a metagenomics approach. PCR-based method is usually used to detect and confirm the presence or absence of a toxin gene in targeted foodborne bacteria [19, 20]. However, metagenomics sequencing techniques as culture-independent methods are currently widely used to identify and characterize microbiota. Therefore, we hypothesized that the metagenomics approach could be used to detect foodborne pathogens in samples such as foods, feces, and soils.

Campylobacteriosis caused mainly by genus *Campylobacter* is a zoonosis of increasing importance in industrialized countries. The most common routes of infection to humans are assumed to be consumption of contaminated raw or undercooked poultry and red meats and contaminated raw milk [21]. At present, more than 90% of Danish human *Campylobacter* cases are caused by *C. jejuni*, whereas approximately 5% of cases are diagnosed as *C. coli* [22]. National monitoring for antimicrobial resistance in Denmark has shown that the prevalence of *C. jejuni* is between 3 and 6% in finisher pigs over the past 5 years ( 1999 ~ 2003 ) [23, 24]. However, other studies conducted in the United States, Canada, and Europe have shown that 30 to 50% of *Campylobacter* isolates found in pigs and pork are *C. jejuni* [25-29]. *Campylobacter* was detected in cattle and pig feces in the present study, with pig feces having higher relative abundance of *Campylobacter* than cattle feces. Thus, we should be aware of the potential contamination of this pathogen in beef and pork products.

Moreover, *Clostridium* was detected in feces of three different species in our study. *Clostridium perfringens* is found in the environment. It can cause illness in both humans and animals. *C. perfringens* is found in soil, water from rivers and sewage, intestines, and feces of mammals including humans. Symptoms caused by *C. perfringens* include diarrhea and stomach cramps within 6~24 hours after infection [30]. *C. perfringens* causes foodborne illness through producing toxins of five types (A, B, C, D, and E). Type A is associated with foodborne pathogens in humans. It produces *Clostridium perfringens* alpha toxin (CPA) along with enterotoxin (CPE) with or without *Clostridium perfringens* beta 2 toxin (CPB2) [31, 32]. It has been reported that *C. perfringens* is readily detected in foods such as pork, beef, chicken, and sheep [33-35]. In this study, the relative abundance of *C. perfringens* in chicken feces was the highest among three different livestock species. *C. perfringens* has been frequently detected in the intestinal tract and processed poultry meat. When intestinal contents of broiler chickens were analyzed for the presence of *C. perfringens*, approximately 75% to 95% of animals were found to be positive [36]. Furthermore, a previous report from Iran has shown that *cpe* gene of *C. perfringens* was founded in 27% and 32.76% intestinal contents of tested cattle and poultry, respectively [37].

*Staphylococcus aureus* is one of main public health concerns worldwide. It is a dangerous human pathogen that causes Staphylococcal food-borne disease (SFD) [38-40]. The virulence of *S. aureus* is mainly related to the production of a variety of protein toxins. The production of enterotoxins is associated with sepsis-related infections, food poisoning , pneumonia, and toxic shock syndrome [41]. It has been shown that staphylococcal enterotoxins (SEs) take an important role in the manifestation of a variety of human diseases [42, 43]. Chicken is the most common source of *S. aureus* in the world including South Korea. In this study, *Staphylococcus* was also detected in cattle, chicken, and pig samples at the genus level, with chicken feces having the highest relative abundance of those genera.

In addition, potential pathogens such as *Helicobacter* and *Pseudomonsas* were also identified in this study. The relative abundance of *Helicobacter* was the highest in pig feces and the relative abundance of *Pseudomonsas* was high in chicken and cattle fecal samples used in this study.

Helicobacter is a Gram-negative bacterium with a helical shape [44]. *Helicobacter pylori* is the most important pathogen that causes gastric diseases in humans. However, non-*Helicobacter pylori* helicobacter species could cause a disease in human as well as several animals [45]. *Helicobacter* species are commonly found in pig stomach [46].

Genus *Pseudomonas* contains 191 known species [47]. Certain *Pseudomonas* species including *P. syringae*, *P. oryzihabitans*, and *P. aeruginosa* can cause diseases in humans at hospital environments and animals [48-50]. Nosocomial infections of *Pseudomonas* can cause gastrointestinal infections and bacteremia [51]. Most *Pseudomonas* can produce polysaccharide which is associated with biofilm formation, resulting in resistance to phagocytosis by white blood cells. Most *Pseudomonas* are also naturally resistant to the majority of related beta-lactam antibiotics and penicillin [52].

Overall, we showed that microbiota of cattle, chicken, and pig feces were significantly different, and the distribution of foodborne pathogens in different livestock feces was also clearly different. Our results shall shed a new light on the food-borne risk assessments of livestock using metagenomics even with the limitation. Nevertheless, the accumulative and systematic analyses will help us to obtain a more reliable and general tendency in bacterial contamination in livestock feces and the further validation tests, such as isolation and identification of pathogens using selective medium or real-time PCR would be essential to present more reliable results from the metagenomics study. Even though identification of foodborne pathogens at genus level is limited, this study highlights the feasibility of using metagenomics approach to assess foodborne risk of livestock feces using metagenomics even with the limitation. However, the further studies using whole metagenome shotgun sequencing to detect toxin genes would warrant the identification of pathogenic traits in foodborne pathogens

## Supplemental Materials

Supplementary data for this paper are available on-line only at http://jmb.or.kr.

## Figures and Tables

**Table 1:** Average values of Alpha-diversity measurements for feces of livestock.

	Observed OTUs	Chao	Shannon	Simpson
Cattle	20442.76	82076.52	11.33	1
Chicken	4536.545	13068.23	5.984739	0.93
Pig	6903.412	23217.42	7.170497	0.95
