# Supplementary material for: Evaluating the Prevalence of Foodborne Pathogens in Livestock Using Metagenomics Approach
Source: J Microbiol Biotechnol. 2021 Oct 15;31(12):1701–8. doi: 10.4014/jmb.2109.09038 (PMC9706027; doi:10.4014/jmb.2109.09038)
Supplement: Supplementary file 1 [file jmb-31-12-1701-supple.pdf]

J. Microbiol. Biotechnol.
https://doi.org/10.4014/jmb.2109.09038

Supplementary Table

Supplementary Table 1. The livestock feces sample information

Species	Sample	location		Sampling date
		latitude	longitude	
Cattle	Feces	36.887637	126.956768	2019.11
	Feces	36.824100	128.561473	2020.02
	Feces	36.730063	126.861836	2020.08
Chicken	Feces	36.680083	127.196251	2019.09
	Feces	35.888365	127.102145	2020.02
	Feces	36.368967	127.349615	2020.07
Pig	Feces	36.812864	127.613446	2020.01
	Feces	34.594361	126.391230	2020.01
	feces	36.523742	126.708225	2020.06

Supplementary Table 2. Number of 16S rRNA gene sequence reads of livestock fecal microbiota before and after quality control.

Sample ID	Number of sequence reads		QC-passed Reads, %
	Pre-QC	Post-QC	
CATTLE1	137,157	135,383	98.71
CATTLE2	126,376	124,092	98.19
CATTLE3	149,978	147,492	98.34
CATTLE4	132,065	129,602	98.14
CATTLE5	119,262	117,390	98.43
CATTLE6	136,881	134,591	98.33
CATTLE7	138,283	135,941	98.31
CATTLE8	133,450	130,481	97.78
CATTLE9	132,101	130,033	98.43
CATTLE10	116,910	114,843	98.23
CATTLE11	142,614	140,289	98.37
CATTLE12	142,523	139,621	97.96
CATTLE13	150,918	148,484	98.39
CATTLE14	133,453	131,510	98.54
CATTLE15	150,161	147,924	98.51
CATTLE16	170,640	168,444	98.71
CATTLE17	140,730	138,572	98.47
CATTLE18	164,461	161,649	98.29
CATTLE19	153,883	151,175	98.24
CATTLE20	138,073	135,351	98.03
CATTLE21	98,891	96,224	97.30
CATTLE22	84,791	83,030	97.92
CATTLE23	87,918	86,054	97.88
CATTLE24	105,743	103,352	97.74
CATTLE25	93,711	91,275	97.40
CATTLE26	86,386	84,562	97.89
CATTLE27	82,116	80,091	97.53
CATTLE28	97,587	95,421	97.78
CATTLE29	98,424	96,545	98.09
CATTLE30	81,590	79,677	97.66
CATTLE31	91,281	89,115	97.63
CATTLE32	71,179	69,617	97.81
CATTLE33	86,677	84,408	97.38
CHICKEN1	127,440	125,365	98.37
CHICKEN2	139,030	137,414	98.84
CHICKEN3	135,126	133,165	98.55
CHICKEN4	157,691	154,965	98.27
CHICKEN5	158,185	155,817	98.50
CHICKEN6	145,078	143,236	98.73
CHICKEN7	168,995	166,707	98.65
CHICKEN8	154,718	151,913	98.19
CHICKEN9	142,117	139,843	98.40
CHICKEN10	142,042	139,797	98.42

CHICKEN11	149,453	147,731	98.85
CHICKEN12	133,994	132,229	98.68
CHICKEN13	137,098	135,141	98.57
CHICKEN14	145,615	143,211	98.35
CHICKEN15	133,665	131,560	98.43
CHICKEN16	132,263	129,738	98.09
CHICKEN17	163,906	161,607	98.60
CHICKEN18	146,472	144,375	98.57
CHICKEN19	145,799	143,519	98.44
CHICKEN20	154,075	151,624	98.41
CHICKEN21	92,911	90,517	97.42
CHICKEN22	88,304	86,288	97.72
CHICKEN23	100,964	98,455	97.51
CHICKEN24	103,024	100,601	97.65
CHICKEN25	88,032	86,221	97.94
CHICKEN26	82,935	81,138	97.83
CHICKEN27	107,335	104,869	97.70
CHICKEN28	75,027	73,191	97.55
CHICKEN29	81,671	79,902	97.83
CHICKEN30	94,595	92,680	97.98
CHICKEN31	94,449	91,920	97.32
CHICKEN32	96,432	93,926	97.40
CHICKEN33	94,028	91,784	97.61
PIG1	142,119	139,159	97.92
PIG2	138,695	135,978	98.04
PIG3	142,050	139,573	98.26
PIG4	150,367	147,240	97.92
PIG5	152,449	149,484	98.06
PIG6	153,428	151,371	98.66
PIG7	133,533	131,576	98.53
PIG8	148,795	147,132	98.88
PIG9	158,616	156,010	98.36
PIG10	153,278	151,016	98.52
PIG11	154,954	152,117	98.17
PIG12	145,246	142,977	98.44
PIG13	155,917	152,952	98.10
PIG14	136,740	134,666	98.48
PIG15	125,904	123,617	98.18
PIG16	140,935	138,607	98.35
PIG17	135,799	133,482	98.29
PIG18	132,015	129,951	98.44
PIG19	127,231	124,864	98.14
PIG20	143,524	141,036	98.27
PIG21	114,604	104,669	91.33
PIG22	130,469	118,731	91.00
PIG23	198,545	181,204	91.27
PIG24	163,384	148,793	91.07
PIG25	177,876	162,561	91.39

PIG26	210,509	191,371	90.91
PIG27	121,557	110,972	91.29
PIG28	165,995	150,273	90.53
PIG29	154,125	141,327	91.70
PIG30	152,049	138,606	91.16
PIG31	168,255	154,609	91.89
PIG32	132,523	121,411	91.62
PIG33	168,232	155,251	92.28
PIG34	130,404	119,323	91.50
